# Reduced Glutathione Promoted Growth Performance by Improving the Jejunal Barrier, Antioxidant Function, and Altering Proteomics of Weaned Piglets

**DOI:** 10.3390/antiox14010107

**Published:** 2025-01-17

**Authors:** Zhimei Tian, Yiyan Cui, Miao Yu, Dun Deng, Zhenming Li, Xianyong Ma, Mingren Qu

**Affiliations:** 1 Jiangxi Province Key Laboratory of Animal Nutrition, Animal Nutrition and Feed Safety Innovation Team, College of Animal Science and Technology, Jiangxi Agricultural University, Nanchang 330006, China; tianzhimei@gdaas.cn; 2 State Key Laboratory of Swine and Poultry Breeding Industry, Key Laboratory of Animal Nutrition and Feed Science in South China, Ministry of Agriculture and Rural Affairs, Guangdong Provincial Key Laboratory of Animal Breeding and Nutrition, Institute of Animal Science, Guangdong Academy of Agricultural Sciences, Guangzhou 510640, China

**Keywords:** reduced glutathione, growth performance, jejunal barrier, proteomics, weaned piglets

## Abstract

Reduced glutathione (GSH) is a main nonenzymatic antioxidant, but its effects and underlying mechanisms on growth and intestinal health in weaned piglets still require further assessment. A total of 180 weaned piglets were randomly allotted to 5 groups: a basal diet (CON), and a basal diet supplemented with antibiotic chlortetracycline (ABX), 50 (GSH1), 65 (GSH2), or 100 mg/kg GSH (GSH3). Results revealed that dietary GSH1, GSH2, and ABX improved body weight and the average daily gain of weaned piglets, and ABX decreased albumin content but increased aspartate aminotransferase (AST) activity and the ratio of AST to alanine transaminase levels in plasma. GSH2 significantly decreased glucose content but increased the content of triglyceride and cholesterol in the plasma. Both GSH1 and GSH2 improved the jejunal mucosa architecture (villus height, crypt depth, and the ratio of villus height to crypt depth), tight junction protein (*ZO*-1 and *Occludin*), and antioxidant capacity (CAT and MDA), and the effects were superior to ABX. Dietary GSH improved the jejunal barrier by probably inhibiting the myosin light chain kinas pathway to up-regulate the transcript expression of tight junction protein (*ZO*-1 and *Occludin*) and Mucins. Through the proteomics analysis of the jejunal mucosa using 4D-DIA, the KEGG pathway enrichment analysis showed that differentiated proteins were significantly enriched in redox homeostasis-related pathways such as glutathione metabolism, cytochrome P450, the reactive oxygen species metabolic pathway, the oxidative phosphorylation pathway, and the phosphatidylinositol 3-kinase-serine/threonine kinase pathway in GSH2 vs. CON and in GSH2 vs. ABX. The results of proteomics and qRT-PCR showed that GSH supplementation might dose-dependently promote growth performance and that it alleviated the weaning stress-induced oxidative injury of the jejunal mucosa in piglets by activating SIRTI and Akt pathways to regulate GPX4, HSP70, FoxO1. Therefore, diets supplemented with 50–65 mg/kg GSH can promote the growth of and relieve intestinal oxidative injury in weaned piglets.

## 1. Introduction

Weaning is a critical stage and a bottleneck for piglet production. Owing to alterations in nutrition, the immune system, faming conditions, and psychology, the early weaning of piglets generally triggers oxidative stress, intestinal dysfunction, and growth restriction [1,2]. Antibiotics, especially the broad-spectrum antibiotic chlortetracycline (alias aureomycin), were widely used to promote growth, the rate of gain, and feed efficiency in weaned piglets [3,4,5,6]; however, they have been forbidden in the European Union since 2006 and in China since 2020 due to their side-effects on animals, the ecological environment, and microbial resistance. Many strategies as antibiotic alternatives have been implemented to relieve intestinal redox imbalance and promote the growth of weaned piglets.

Natural antioxidants such as polyphenols, polysaccharide, and Chinese herbs have been investigated for application in promoting growth and alleviating oxidative stress in weaned piglets [7,8,9]. GSH is a main nonenzymatic antioxidant for maintaining cellular redox homeostasis and detoxifying electrophiles [10]. Under normal physiological conditions, cells need a high GSH concentration for maintaining redox homeostasis [11]. Weaning-induced intestinal oxidation and inflammation typically necessitates a large amount of GSH to eliminate reactive oxygen species (ROS) production and interrupts the intestinal redox balance, which results in GSH dysregulation in the intestine of weaned piglets [12]. Therefore, we inferred that exogenous GSH supplementation can alleviates weaning-induced gut damage and growth restriction.

GSH has the potential to alleviate oxidative stress-induced diseases such as diabetic nephropathy, parkinsonism, COVID-19, and steatosis [13,14,15,16]; therefore, GSH has been applied as an antioxidative and antiaging drug in humans [17]. Previous studies found that GSH-supplementation in freezing extenders counteracted cryopreservation-related decrease in the acrosin activity of boar spermatozoa [18] and improved bovine embryo development through the redox regulation of an increase in intracellular GSH levels by the γ-glutamyl cycle and ROS elimination [19]. Dietary GSH alleviated toxic intestinal injury in piglets [20,21] and promoted the growth as well as the intestinal health of juvenile triploid *Oncorhynchus mykiss* [22]. However, few studies have investigated the application of GSH as the feed additive and the mechanism of GSH on growth and intestinal health in weaned piglets still requires further assessment. Therefore, the objective of the present study was to explore whether GSH can replace chlortetracycline to promote growth performance and alleviate intestinal oxidative injury in weaned piglets and to further illustrate its underlying mechanism. This study will provide the application strategy of GSH as the antibiotic alternative and the growth promoter for enhancing the intestinal health of weaned piglets.

## 2. Materials and Methods

### 2.1. Animal Ethics Statement

Animal procedures and experiments were approved by the Animal Care and Use Committee of Guangdong Academy of Agricultural Science (No. 2023005) and conducted according to the Guide for Care and Use of Laboratory Animals of the National Research Council of China.

### 2.2. Glutathione and Chlortetracycline

GSH extracted from yeast was provided by Shandong Jincheng Biological Pharmaceutical Co., Ltd. (Zibo, Shandong, China). The chlortetracycline was purchased from Guangdong Newland Feed Science and Technology Co., Ltd. (Guangzhou, Guangdong, China).

### 2.3. Animals and Experimental Design

A total of 180 weaned barrow piglets (Duroc × Landrace × Yorkshire) aged at 21 d with similar body weights (BW, 6.63 ± 0.04 kg), were randomly allotted to 5 dietary treatments: (1) Negative control (CON, the basal diet); (2) Antibiotic—Positive control (ABX, the basal diet supplemented with 75 mg/kg chlortetracycline); (3) 50 mg/kg GSH (GSH1, the basal diet supplemented with 50 mg/kg GSH); (4) 65 mg/kg GSH (GSH2, the basal diet supplemented with 65 mg/kg GSH); (5) 100 mg/kg GSH (GSH3, the basal diet supplemented with 100 mg/kg GSH). A total of 36 piglets per dietary treatment were assigned to 6 replicate pens with 6 piglets per pen. The basal diet (Appendix A: Table A1) was formulated to meet nutrient recommendations of weaned piglets based on NRC (2012) [23]. Piglets were housed in high bed pens (4.54 by 1.64 m, 1.24 m^2^ of pen floor space per piglet) equipped with a sided feeder and a nipple watering device and had free access to feed and water throughout the 28 d experimental period.

### 2.4. Growth Performance

Piglets were weighed at the beginning, at 14 d, and at the end of the trial after fasting for 12 h. Feed intake per pen was recorded daily. The average daily gain (ADG), average daily feed intake (ADFI), and F/G (feed to gain ratio) were evaluated based on the BW and feed intake of the piglets.

### 2.5. Slaughter and Tissue Sample Collection

Pigs aged 49 d were fasted for 12 h at the end of the animal experiment, one piglet per pen was randomly selected for plasma collection by jugular venipuncture using a 10 mL heparin-coated vacuum, and then the blood samples were subsequently centrifuged at 3000× g/min for 15 min at 4 °C for separating plasma. The plasma was aliquoted into 4 sterile Eppendorf tubes and these were stored at −80 °C until measurement. After blood sampling, piglets were euthanized after anaesthetization and exsanguination for samples collection. The jejunum was immediately dissected and an about 1 cm length segment of mid-jejunum per piglet was fixed in 10% formalin solution for morphological analysis. After cutting longitudinally, emptying contents, and flushing with cold PBS, mid-jejunal mucosa was scraped and collected with sterile glass slides into 3 sterile Eppendorf tubes, and was frozen in liquid nitrogen and then held at −80 °C before examination.

### 2.6. Histochemistry Staining

The jejunal segments after fixing were used for morphology analysis using hematoxylin and eosin staining protocol as performed our previous study [24]. The morphometry of the jejunal mucosa was detected using Pannoramic P250 FLSAH (3DHISTECH, Inc. Budapest, Hungary). Villi height (VH) and crypt depth (CD) were then measured using CaseViewer 3.3 software (JAVS, Inc. Louisville, KY, USA).

### 2.7. Blood Biochemistry Index and Redox Capacity of the Jejunal Mucosa

Plasma biochemical parameters and the jejunal mucosa antioxidant capacity were detected using the respective kits (Nanjing Jiancheng, Nanjing, China), following the manufacture’s protocols. The jejunal mucosa was homogenized with 0.9% NaCl as a 1:9 (*w*/*v*) ratio and centrifuged at 3500× g at 4 °C for 15 min. Supernatant was collected to detect the content of malondialdehyde (MDA), total antioxidant capacity (T-AOC), and activities of antioxidant enzymes like total superoxide dismutase (T-SOD), glutathione peroxidase (GPX), and catalase (CAT).

### 2.8. Proteomics Analysis of the Jejunal Mucosa Through Four-Dimensional Data-Independent Acquisition (4D-DIA)–Mass Spectrometry (MS)

Samples were first ground in liquid nitrogen, and six samples of the jejunal mucosa (100 mg) per group were pooled into three mixed samples. Samples were sonicated three times on ice by using a high-intensity ultrasonic processor in a lysis buffer (8 M urea including 1 mM of PMSF and 2 mM of EDTA). The remaining debris were removed through centrifugation at 15,000× *g* at 4 °C for 10 min. Finally, the protein concentration was determined using the BCA kit (Thermo Fisher Scientific Inc., Waltham, MA, USA) according to the manufacturer’s instructions.

Equal amounts of proteins from each sample were used for trypsin digestion. Following trypsin digestion, peptides were desalted using the C18 cartridge, followed by drying with a vacuum concentration meter. Then, the peptides were concentrated through vacuum centrifugation and redissolved in 0.1% (*v*/*v*) formic acid. Approximately 200 ng of peptides were separated within 60 min at a flow rate of 0.3 µL/min on a commercially available reverse-phase C18 column with an integrated Captive Spray Emitter (25 cm × 75 μm ID, 1.6 μm, Aurora Series) with a CaptiveSpray nano-electrospray ion source (CSI, IonOpticks Inc., Melbourne, Australia) by using a nanoElute UHPLC (Bruker Daltonics, Inc., Rheinstetten, Germany). For liquid chromatography–mass spectrometry (LC-MS)/MS analysis, mobile phases A and B were produced with 0.1% formic acid in water and 0.1% formic acid in can, respectively. Mobile phase B was increased from 2% to 22% over the first 45 min, increased to 35% over the next 5 min, further increased to 80% over the next 5 min, and then held at 80% for 5 min. The LC was coupled online to a hybrid timsTOF Pro2 (Bruker Daltonics, Rheinstetten, Germany) through a CSI. The timsTOF Pro2 was operated in a Data-Dependent Parallel Accumulation–Serial Fragmentation mode with 10 PASEF MS/MS frames in 1 complete frame. MS raw data were analyzed using DIA-NN (v1.8.1) in a library-free method. The false discovery rate of search results was adjusted to <1% at both protein and precursor ion levels, and the remaining identifications were used for further quantification analysis.

### 2.9. Quantitative Real-Time PCR (qRT-PCR)

Total RNA of the jejunal mucosa sample was extracted and reverse-transcribed to cDNA according to the manufacturer’s protocols of the kit (Takara Bio Inc., Ostu, Japan). qRT-PCR (Bio-Rad System) was performed and analyzed according to our previous protocol [24]. The primer sequences for qRT-PCR are shown in Table A2 (Appendix B).

### 2.10. Statistical Analysis

Using GraphPad Prism 9 (GraphPad Software, Inc. San Diego, CA, USA), data except proteomics data were analyzed through one-way analysis of variance (ANOVA), followed by Tukey’s post hoc test. The linear and quadratic contrasts were tested using orthogonal polynomial contrasts, and coefficients of orthogonal polynomial contrasts were corrected and determined for unequal spaced dilutions and compost inclusion rates based on the procedure described by St. Martin [25]. Differences were identified as statistically significant at *p* ≤ 0.05. The results were presented as means and standard error of the mean (SEM). In the proteomics analysis of this study, differentially expressed proteins were filtered using Student’s *t*-test at FC > 1.5 or FC < 0.67, and *p* < 0.05. For bioinformatics analysis, annotation, functional enrichment, and enrichment-based clustering were performed based on the Kyoto encyclopedia of genes and genomes (KEGG). The KEGG was used to identify enriched pathways by a two-tailed Fisher’s exact test to evaluate the enrichment of differentially expressed proteins against all identified proteins.

## 3. Results

### 3.1. Effects of Dietary GSH on Growth Performance in Weaned Piglets

Dietary ABX, GSH1, and GSH2 significantly improved the BW (35 d and 49 d), and the ADG at 1–14 days, 15–28 days, and 1–28 days of weaned piglets compared with CON (*p* < 0.05, Table 1). Dietary GSH2 improved ADFI at 1–28 days compared with CON and GSH3 (*p* < 0.05). Dietary GSH3 had no significant effects on BW (body weight), ADG, ADFI, and F/G compared with other diets (*p* > 0.05). No significant differences were observed in ADFI at 1–14 days and 15–28 days and F/G among the five groups (*p* > 0.05).

### 3.2. Effects of Dietary GSH on Biochemical Parameters of Plasma in Weaned Piglets

As shown in Table 2, piglets fed ABX had lower albumin but higher aminotransferase (AST) and a higher AST to alanine transaminase (ALT) ratio (AST/ALT) compared with CON (*p* < 0.05). Piglets fed GSH1 had lower AST and AST/ALT compared with ABX (*p* < 0.05) but no significant differences were observed in these biochemical parameters compared with CON, GSH2, and GSH3 (*p* > 0.05). Piglets fed GSH2 had higher triglyceride and cholesterol but lower glucose in plasma compared with CON (*p* < 0.05), and had higher albumin but lower AST and AST/ALT compared with ABX (*p* < 0.05). Pigs fed GSH3 had lower albumin compared with CON but lower AST and AST/ALT compared with CON and ABX (*p* < 0.05), as well as higher glucose but lower albumin and urea nitrogen compared with GSH2 (*p* < 0.05).

### 3.3. Effects of Dietary GSH on Histomorphology and Epithelial Barrier of the Jejunal Mucosa in Weaned Piglets

**Figure 1:**
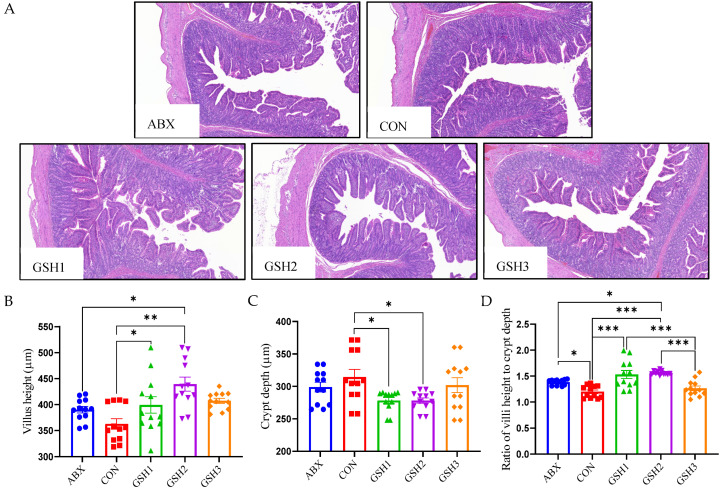
Effect of GSH on histomorphology of the jejunal mucosa in weaned piglets. (**A**) Histology images of jejunum by H&E stains (scale bar, 500 µm). (**B**–**D**) The morphometry of the jejunal mucosa. Note: Values are shown as mean ± SEM. * *p* < 0.05, ** *p* < 0.01, and *** *p* <0.001. ABX, a basal diet supplemented with chlortetracycline; CON, basal diet; GSH1, a basal diet supplemented with 50 mg/kg GSH; GSH2, a basal diet supplemented with 65 mg/kg GSH; GSH3, a basal diet supplemented with 100 mg/kg GSH in the basal diet.

**Figure 2:**
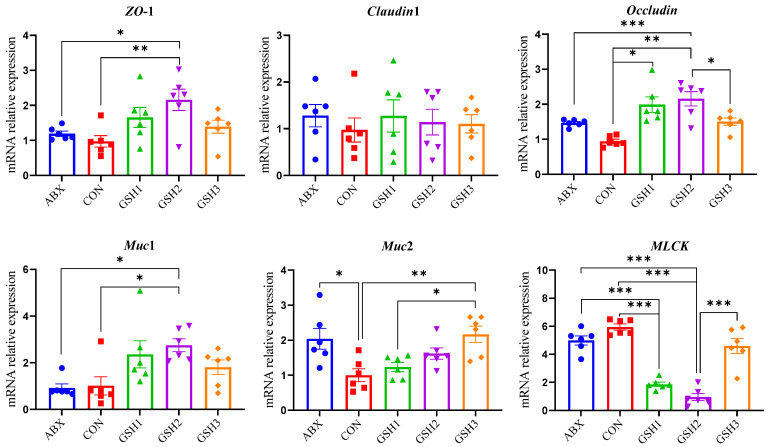
Effect of reduced glutathione on the epithelial barrier of jejunum in weaned piglets. Note: Values are shown as mean ± SEM. * *p* < 0.05, ** *p* < 0.01, and *** *p* <0.001. ABX, a basal diet supplemented with chlortetracycline; CON, basal diet; GSH1, a basal diet supplemented with 50 mg/kg GSH; GSH2, a basal diet supplemented with 65 mg/kg GSH; GSH3, a basal diet supplemented with 100 mg/kg GSH in the basal diet. ZO-1, zonula occludens-1; Muc1/2, mucin 1/2; MLCK, myosin light chain kinas.

The morphology of the jejunal mucosa in Figure 1 showed that piglets fed ABX had a higher ratio of VH to CD (VH/CD) compared with CON but lower VH and VH/CD compared with GSH2 (*p* < 0.05). Piglets fed GSH1 and GSH2 had higher VH and VH/CD but lower CD compared with CON as well as higher VH/CD compared with GSH3 (*p* < 0.05). The transcript abundance of intestinal barrier-related genes in Figure 2 showed that piglets fed ABX had higher *Muc*2 compared with CON (*p* < 0.05). Piglets fed GSH1 had lower myosin light chain kinas (*MLCK*) abundance compared with CON and ABX but higher *occludin* abundance compared with CON as well as lower *Muc*2 abundance compared with GSH3 (*p* < 0.05). Piglets fed GSH2 had a higher abundance of zonula occludens-1 (*ZO*-1), *occludin*, and *Muc*1, but lower *MLCK* abundance compared with CON and ABX (*p* < 0.05). Piglets fed GSH3 had higher *Muc*2 compared with CON but lower *occludin* abundance as well as higher *MLCK* abundance compared with GSH2 (*p* < 0.05).

### 3.4. Jejunal Antioxidant Capacity in Weaned Piglets

**Figure 3:**
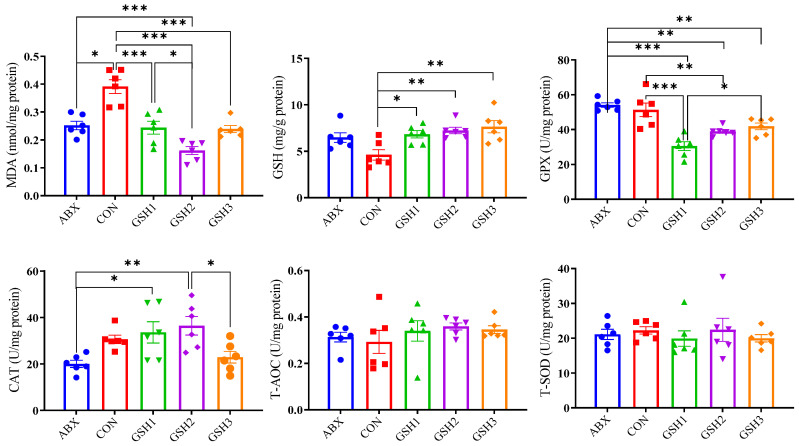
Effects of reduced glutathione on jejunal antioxidant status in weaned piglets. Note: Values are shown as mean ± SEM. * *p* < 0.05, ** *p* < 0.01, and *** *p* <0.001. ABX, a basal diet supplemented with chlortetracycline; CON, basal diet; GSH1, a basal diet supplemented with 50 mg/kg GSH; GSH2, a basal diet supplemented with 65 mg/kg GSH; GSH3, a basal diet supplemented with 100 mg/kg GSH in the basal diet. MDA, malondialdehyde; GSH, reduced glutathione, GPX, glutathione peroxidase; CAT, catalase; T-AOC, total antioxidant capacity; T-SOD, total superoxide dismutase.

Results of oxidant–antioxidant indices in the jejunal mucosa (Figure 3) showed that MDA content was lower in piglets fed ABX, GSH1, GSH2, and GSH3 compared with CON, or in piglets fed GSH2 compared with ABX and GSH1 (*p* < 0.05). Piglets fed GSH1, GSH2, and GSH3 had higher GSH compared with CON (*p* < 0.05). Piglets fed GSH1 and GSH2 had lower GPX but higher CAT compared with ABX (*p* < 0.05). In piglets fed GSH3, GPX was lower compared with ABX but higher compared with GSH1, and CAT was lower compared with GSH2 (*p* < 0.05). No significant differences were observed for T-AOC and T-SOD (*p* > 0.05).

### 3.5. Proteomics and qRT-PCR Analysis Showed Dietary GSH2 Improved the Jejunal Redox Homeostasis of the Jejunal Mucosa in Weaned Piglets

**Figure 4:**
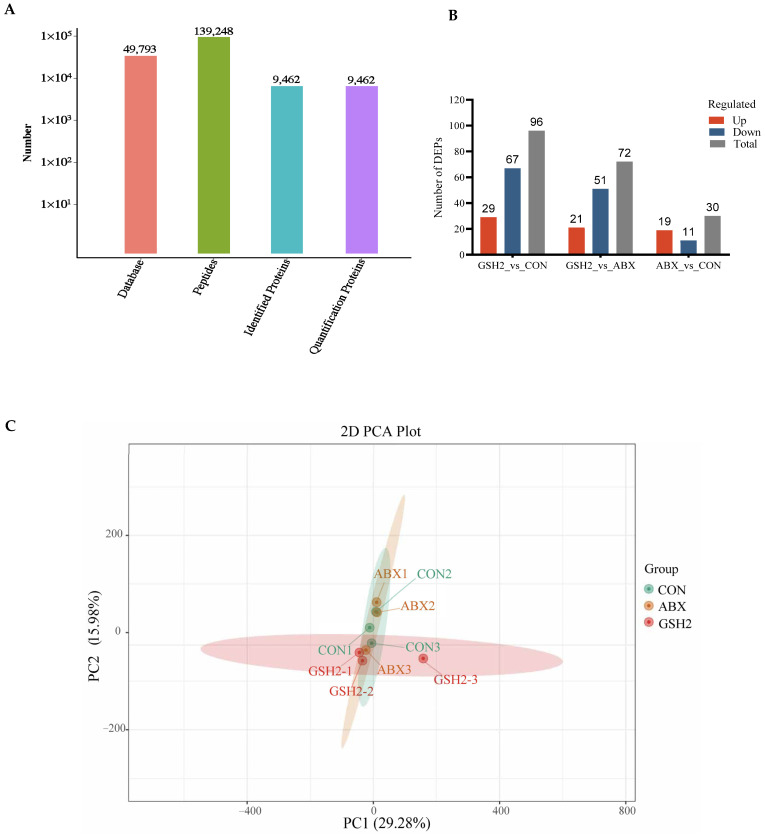

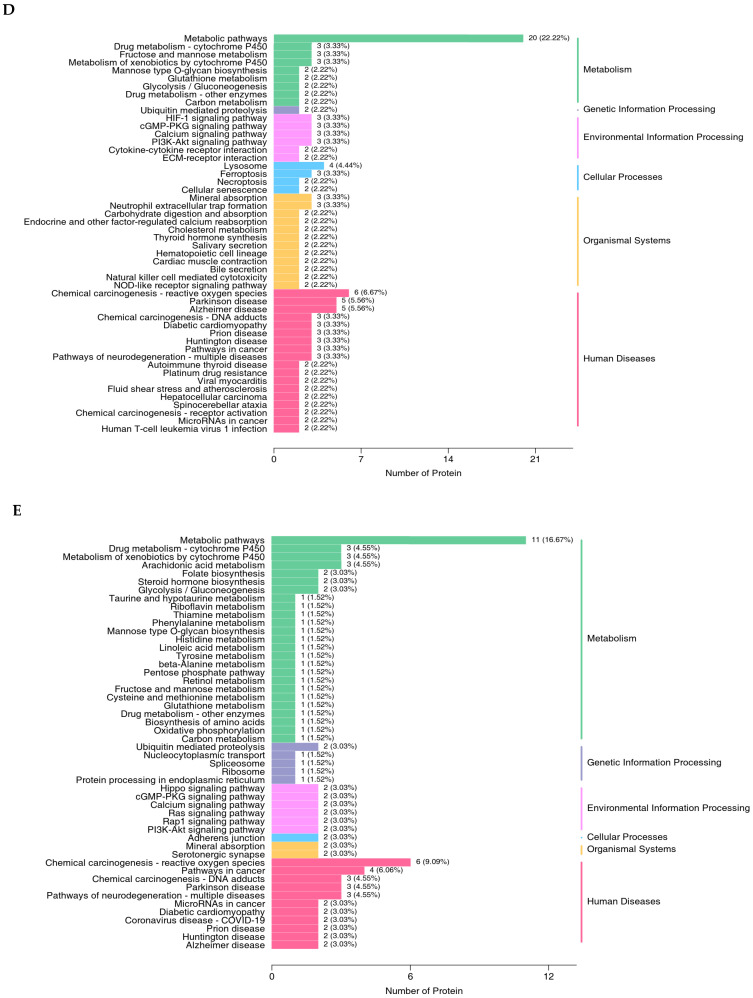

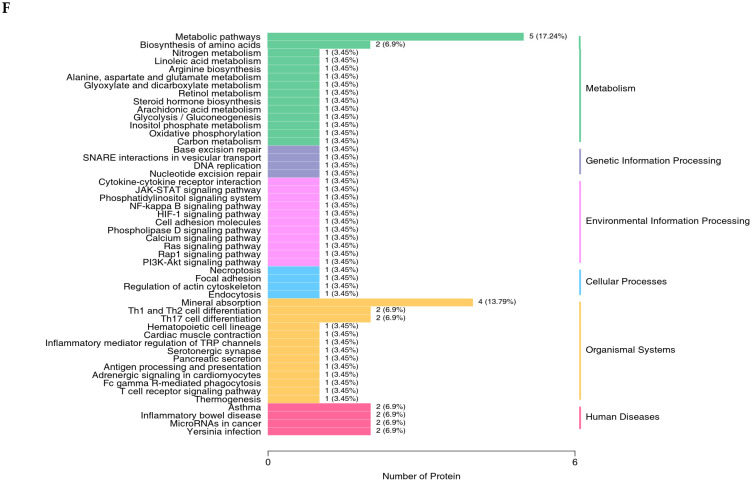
Proteomics analysis of the jejunal mucosa in weaned piglets. (**A**) Qualitative and quantitative analysis of identified proteins; (**B**) Differentially expressed proteins; (**C**) Two-dimensional principal component analysis; (**D**–**F**) KEGG pathway enrichment from GSH2 vs. CON, GSH2 vs. ABX, and ABX vs. CON, respectively. KEGG: Kyoto encyclopedia of genes and genomes. Note: ABX, a basal diet supplemented with chlortetracycline; CON, basal diet; GSH2, a basal diet supplemented with 65 mg/kg GSH in the basal diet.

The proteomics profile of the jejunal mucosa was detected using 4D-DIA methods among the CON, ABX, and the most effective dose of GSH2 groups (Figure 4). A total of 139,248 peptidases were identified, and 9462 proteins were identified from 49,793 proteins of the database (Figure 4A). A total of 96, 72, and 30 differentiated proteins were identified, of which 29, 21, and 19 proteins were up-regulated and 67, 51, and 11 proteins were down-regulated in GSH2 vs. CON, GSH2 vs. ABX, ABX vs. CON, respectively (Figure 4B). The two-dimensional principal component analysis indicated substantially different proteins in the jejunal mucosa among the three groups (Figure 4C). The KEGG pathway enrichment analysis showed that differentiated proteins were significantly enriched in redox homeostasis-related pathways such as glutathione metabolism, cytochrome P450, the ROS metabolic pathway, the oxidative phosphorylation pathway, and the PI3K-Akt (phosphatidylinositol 3-kinase-serine/threonine kinase) pathway in GSH2 vs. CON and in GSH2 vs. ABX (Figure 4D,E). In ABX vs. CON, differentiated proteins were significantly enriched in redox homeostasis-related pathways such as oxidative phosphorylation and the PI3K-Akt pathway (Figure 4F).

**Figure 5:**
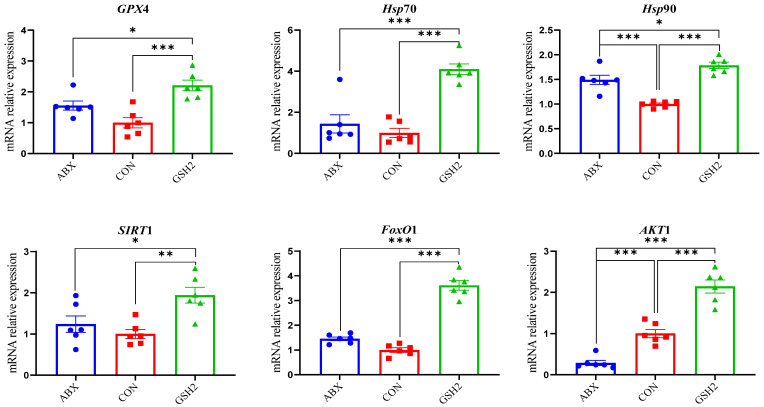
Effect of reduced glutathione on oxidative stress-related genes in the jejunal mucosa of weaned piglets. Note: Values are shown as mean ± SEM. * *p* < 0.05, ** *p* < 0.01, and *** *p* <0.001. ABX, a basal diet supplemented with chlortetracycline; CON, basal diet; GSH1, a basal diet supplemented with 50 mg/kg GSH; GSH2, a basal diet supplemented with 65 mg/kg GSH; GSH3, a basal diet supplemented with 100 mg/kg GSH in the basal diet. GPX4, glutathione peroxidase 4; Hsp70, heat shock protein 70 kDa; Hsp90, heat shock protein 90 kDa; SIRT1, sirtuin 1; FoxO1, forkhead box protein O1; Akt1, serine/threonine kinase 1.

Additionally, differentiated proteins involved in redox homeostasis pathways in Table 3 showed that up-regulated proteins both in GSH2 vs. CON and GSH2 vs. ABX were heat shock protein 90 kDa beta member 1 (Hsp90B1), heat shock protein family A (Hsp70) member 4 (HspA4), glutathione peroxidase 4 (GPX4), sirtuin 1 (SIRT1), forkhead box protein O1 (FoxO1), and serine/threonine kinase 1 (Akt1). However, cytochrome P450 4F3 (CYP4F3) and CYP2C42 were up-regulated but Akt1 was down-regulated in ABX vs. CON. Furthermore, the qRT-PCR results (Figure 5) further verified that GSH2 significantly increased the transcript abundances of *GPX*4, *Hsp*70, *Hsp*90, *SIRT*1, *FoxO*1, and *Akt*1 of the jejunal mucosa compared with CON and ABX (*p* < 0.05). However, ABX increased *Hsp*90 abundance but decreased *Akt*1 abundance compared with CON (*p* < 0.05).

## 4. Discussion

Weaning stress is often accompanied by intestinal redox imbalance, which consequently leads to intestinal damage and growth restriction [1,26]. Weaning-induced oxidative damages of villous architecture, integrity, and redox status affect the intestinal health and growth of piglets [27,28]. Chlortetracycline as a feed additive was used to promote growth performance of weaned piglets [3,4,5,6]; however, it is forbidden due to the side-effects on animals, the ecological environment, and microbial resistance. GSH has diverse benefits of detoxification, redox regulation, and antioxidant protection [10] and has the potential to alleviate oxidative stress-induced diseases [13,14,15,16]. Previous studies reported that GSH can protect against intestinal oxidative injury and growth restriction induced by paraquat- or diquat in weaned piglets [20,21]. In this study, diets supplemented with 50 and 65 mg/kg GSH promoted the growth performance, antioxidant capacity, the jejunal mucosa morphology and barrier function of weaned piglets, and the effects of 65 mg/kg GSH on CAT activity, VH, VH/CD, *ZO*-1, *Occludin*, and *Muc*1 were superior to chlortetracycline. It is therefore indicated that GSH could serve as an effective feed additive, replace chlortetracycline, promote growth, and alleviate the weaning stress of piglets.

Blood biochemical parameters reflect nutrient metabolic characteristics and health status in animals in response to internal or external environments [29]. Results of this study showed that 65 mg/kg GSH supplementation improved plasma triglyceride and cholesterol and reduced glucose compared with CON, which was in agreement with results of previous studies stating that GSH improved triglyceride levels by regulating lipid metabolism [30] and affected glucose levels by regulating glycometabolism [31]. Being the most favorable amino acid source for protein synthesis, albumin is a criterion useful for evaluating the body condition and is related to the immune system [32,33]. Increases in AST and AST/ALT are closely related to liver injury [34]. In this study, 100 mg/kg GSH decreased albumin content compared with CON and 65 mg/kg GSH, while chlortetracycline increased AST and AST/ALT compared with CON and GSH supplementation. Data of this study are in agreement with the results of previous studies, which state that antibiotics might cause liver injury and reduce blood immunity, but that GSH supplementation has advantages over antibiotics, leading to the improvement of nutrient metabolism, body condition, and liver protection [35,36,37].

The intestine is more susceptible to weaning-induced oxidative stress than other tissues, owing to the immature intestinal function, frequent renewal of enterocytes, and continuous exposure to various stimuli from the intestinal tract in weaned piglets [27,38]. The lower CD, and higher VH as well as VH/CD indicate a faster cell maturation and the stronger secretory function of the small intestine [20,39]. In this study, 50 or 65 mg/kg GSH supplementation improved tight junction protein and the jejunal mucosa architecture with higher VH, lower CD, and higher VH/CD, and the effect is superior to that of chlortetracycline. These fundings indicate that exogenous GSH improves intestinal barrier function. Liang et al. [21] found that dietary GSH attenuated diquat-induced intestinal oxidative injury by increasing tight junction protein of *ZO*-1, *occludin*, and *claudin* 1 in weaned piglets. Interestingly, 50 mg/kg GSH increased *occludin* expression compared with CON and 65 mg/kg GSH supplementation improved the abundances of *ZO*-1, *occludin*, and *Muc*1 compared with CON and chlortetracycline. Both chlortetracycline and 100 mg/kg GSH increased *Muc*2 abundance but 100 mg/kg GSH did not affect the jejunal mucosa architecture compared with CON. The intestinal epithelial barrier serves as the first line of defense against microbial and toxin invasion or exposure to foreign antigens by regulating the expression of tight junction proteins and mucins’ secretion. These findings suggest that the appropriate dose of exogenous GSH relieved weaning-induced intestinal barrier injury by improving the tight junction and *Muc*1 to restrict binding, colonization, and the access of pathogens to enterocytes binding, colonization, and the access of pathogens to enterocytes [40,41], which can be fully elucidated through further studies. The MLCK pathway negatively regulates mucin secretion and the expression of tight junction proteins like ZO-1 and occludin [42,43], which was consistent with the effects of GSH in the present study. Therefore, GSH might dose-dependently promote jejunal epithelial barrier function by inhibiting MLCK to promote the expression of the tight junction and mucins.

GSH or its combination with other antioxidants like organic selenium, vitamins, and quercetin ameliorated diquat-, deoxynivalenol-, and zearalenone-induced oxidative stress by decreasing MDA content and by increasing GSH content and SOD activity to enhance the antioxidant capacity in piglets [10,21]. de Oliveira et al. [44] found that treatment with 1% GSH increased GSH content and T-AOC, but did not reduce MDA content and lipoperoxidation in jejunum of rats. However, in the present study, diets supplemented with GSH increased jejunal GSH content and decreased the MDA content as well as the GPX activity of weaned piglets compared with CON. This inconsistency may be attributed to differences in animal species, physiological status, diet, and stress factors. Additionally, the GPX activity was activated and the CAT activity was inhibited with an increase in exogenous GSH, which implies that antioxidants in the body are firstly invoked to clear cellular ROS followed by stimulating antioxidant enzyme systems of the jejunal mucosa when piglets are subjected to weaning-induced oxidative stress [11,12]. Interestingly, chlortetracycline decreased the jejunal mucosa MDA and did not affect antioxidative enzyme systems compared with CON, indicating that chlortetracycline relieves jejunal injury probably by regulating gut microbiota due to its antimicrobial properties [3]. Therefore, the effects of 50 and 65 mg/kg GSH supplementation on the relief of weaning-induced oxidative stress was superior to 100 mg/kg GSH supplementation for weaned piglets.

Proteomics has been applied to identify differential proteins and signal pathways induced in response to different physiological or pathological states [45]. Here, we used an 4D-DIA quantitative proteomic approach to identify differentially expressed proteins in the jejunal mucosa of piglets from the most effective dose of GSH (GSH2), ABX, and CON groups. Regarding the KEGG pathway enrichment, differentiated proteins in response to redox homeostasis were mainly enriched in glutathione metabolism, cytochrome P450, the ROS metabolic pathway, the oxidative phosphorylation pathway, and the PI3K-Akt pathway in GH2 vs. CON or in GSH2 vs. ABX. After analyzing differential proteins and genes related to redox-homeostasis by proteomics and verifying by qRT-PCR, we found that the expression of antioxidant-related proteins and genes such as Hsp90B1/*Hsp*90, HspA4/*Hsp*70, GPX4, SIRT1, FoxO1, and Akt1 was up-regulated in GSH2 vs. CON or GSH2 vs. ABX. Previous studies found that SIRT1 positively regulates Hsp70, Hsp90, and FoxO1 deacetylation against oxidative stress [46,47,48], while antioxidants can attenuate oxidative stress via the SIRT1/FoxO1 and PI3K/Akt signaling pathways [49,50]. GSH and its precursor substance, cysteine, induced GPX4 synthesis and the activation of the PI3K/Akt pathway which attenuates cell injury via enhancing GPX4 expression [51,52,53]. Moreover, GSH/GPX4 and HSPA5/GPX4 were involved in regulating oxidative stress [54,55]. Therefore, exogenous GSH might ameliorate the jejunal oxidative damage of weaned piglets via the PI3K/Akt pathway to enhance GPX4 expression and GSH metabolism as well as via SIRT1 to regulate FoxO1, Hsp70, and Hsp90. Furthermore, the GSH precursors cysteine, glutamate, and glycine exhibit antioxidant function [56,57]. The KEGG pathway analysis revealed that GSH metabolism was enriched in GH2 vs. CON. Therefore, in addition to GSH itself, its precursors produced by dietary GSH also play a role in alleviating oxidative stress in the jejunal mucosa of weaned piglets. Additionally, Wu et al. [58] discovered that SIRT1 inhibited the MLCK pathway by mediating deacetylation, which was consistent with the tendency of SIRT1 and MLCK in this study, indicating that dietary GSH might promote the expression of the tight junction and Muc1 by SIRT1 to inhibit the MLCK pathway.

In this study, differentiated proteins in response to redox homeostasis were mainly enriched in the oxidative phosphorylation and PI3K-Akt pathway in ABX vs. CON. Furthermore, Akt expression was down-regulated and the expression of CYP2C42 and CYP4F3 expression was up-regulated in ABX vs. CON, suggesting that antibiotics probably inhibited Akt activation and induced CYP expression and resulted in gut microbiota depletion and anti-inflammation by increasing CYP4F3 and CYP2C [59,60,61]. However, increased Hsp90 can relieve stress-induced endothelial cells’ injury by activating Akt [62], which is inconsistent with the effects of chlortetracycline in this study. This inconsistency may be attributed to the fact that chlortetracycline improves Hsp90 expression, thereby probably resulting in the proteasomal degradation of Akt rather than regulating its conformational maturation [61], a hypothesis which can be examined through further studies.

## 5. Conclusions

The supplementation with an optimal dose of GSH (65 mg/kg) promoted growth performance, jejunal barrier function, and antioxidant function, and the effects on CAT activity, VH, VH/CD, *ZO*-1, *Occludin*, and *Muc*1 are superior to chlortetracycline. The effects of GSH on antioxidant function might be attributed to alleviating GSH dysregulation and the activation of SIRTI and Akt pathways to regulate their downstream target genes GPX4, Hsp, FoxO1, and MLCK in jejunal mucosa. Therefore, GSH can replace antibiotics in feedstuffs for alleviating weaning-induced intestinal oxidative injury and the growth restriction of piglets. However, the differentiated mechanism between GSH and chlortetracycline on antioxidant function needs to be confirmed by microbiomics.

## Figures and Tables

**Table 1:** Effects of reduced glutathione on growth performance of weaned piglets.

Item	Treatments ^B^					SEM	*p*-Value ^A^		
	ABX	CON	GSH1	GSH2	GSH3		ANOVA	Linear	Quadratic
Body weight, kg									
21 d	6.63	6.63	6.63	6.63	6.63	0.01	0.968	0.981	0.98
35 d	10.87 ^a^	10.26 ^b^	10.82 ^a^	10.88 ^a^	10.35 ^ab^	0.13	0.030	0.038	0.872
49 d	16.38 ^a^	15.01 ^c^	16.11 ^ab^	16.65 ^a^	15.27 ^bc^	0.28	0.007	0.010	0.830
Days 1 to 14									
ADG, g	302.77 ^a^	259.13 ^b^	299.17 ^a^	303.55 ^a^	265.20 ^ab^	9.24	0.001	0.005	0.836
ADFI, g	373.97	362.72	387.63	397.81	369.15	6.38	0.139	0.016	0.591
F/G, g/g	1.24	1.42	1.31	1.31	1.41	0.03	0.074	0.352	0.648
Days 15 to 28									
ADG, g	393.30 ^a^	339.51 ^b^	378.13 ^a^	411.83 ^a^	351.79 ^ab^	12.35	0.001	0.002	0.546
ADFI, g	555.52	532.16	568.50	595.00	539.33	11.18	0.137	0.022	0.455
F/G, g/g	1.42	1.57	1.51	1.46	1.57	0.02	0.361	0.517	0.971
Days 1 to 28									
ADG, g	348.04 ^a^	299.327 ^b^	338.65 ^a^	357.69 ^a^	308.496 ^ab^	10.14	0.001	0.002	0.785
ADFI, g	464.753 ^ab^	447.44 ^b^	478.07 ^ab^	496.41 ^a^	454.24 ^b^	8.75	0.048	0.006	0.422
F/G, g/g	1.34	1.50	1.42	1.39	1.49	0.02	0.078	0.335	0.872

^a, b, c^ Means in a row without a common superscript differ at *p* < 0.05; ^A^ *p* values indicate the effects of GSH by ANOVA and contrasts (linear and quadratic) analyses, respectively. ^B^ ABX, a basal diet supplemented with chlortetracycline; CON, basal diet; GSH1, a basal diet supplemented with 50 mg/kg GSH; GSH2, a basal diet supplemented with 65 mg/kg GSH; GSH3, a basal diet supplemented with 100 mg/kg GSH in the basal diet. ADG, average daily gain; ADFI, average daily feed intake; F/G, feed to gain ratio; ANOVA, one-way analysis of variance.

**Table 2:** Effects of GSH on biochemical indexes in plasma of weaned piglets.

Item	Treatments ^B^					SEM	*p*-Value ^A^		
	ABX	CON	GSH1	GSH2	GSH3		ANOVA	Linear	Quadratic
Triglyceride (mmol/L)	0.35 ^ab^	0.30 ^b^	0.36 ^ab^	0.48 ^a^	0.32 ^ab^	0.03	0.034	0.006	0.155
Cholesterol (mmol//L)	2.12 ^ab^	1.91 ^b^	2.04 ^ab^	2.53 ^a^	2.12 ^ab^	0.10	0.062	0.018	0.387
LDLC (mmol//L)	1.77	1.61	1.64	1.49	1.68	0.05	0.498	0.278	0.186
HDLC (mmol//L)	0.82	0.96	0.77	0.91	0.82	0.04	0.491	0.556	0.114
Glucose (mM/L)	3.84 ^ab^	4.51 ^a^	3.77 ^ab^	3.26 ^b^	4.30 ^a^	0.22	0.001	<0.001	0.365
Total protein (g/L)	49.36	49.82	50.33	53.56	49.91	0.76	0.467	0.142	0.283
Albumin (g/L)	32.98 ^b^	38.62 ^a^	34.53 ^ab^	39.00 ^a^	32.09 ^b^	1.43	0.006	0.63	<0.001
Urea nitrogen (mmol/L)	14.27 ^ab^	13.02 ^ab^	15.74 ^ab^	17.03 ^a^	11.62 ^b^	0.96	0.016	0.012	0.110
AST (U/L)	36.14 ^a^	20.03 ^b^	11.75 ^bc^	17.34 ^bc^	7.14 ^c^	4.95	<0.001	<0.001	0.163
ALT (U/L)	17.09	17.75	17.69	18.60	15.86	0.45	0.714	0.607	0.244
AST/ALT	2.28 ^a^	1.15 ^b^	0.66 ^bc^	0.94 ^bc^	0.46 ^c^	0.31	<0.001	0.001	<0.001
AKP (U/L)	168.32	174.38	175.18	184.35	156.66	4.57	0.675	0.530	0.235

^a, b, c^ Means in a row without a common superscript differ at *p* < 0.05; ^A^ *p* values indicate the effects of GSH by ANOVA and contrasts (linear and quadratic) analyses, respectively. ^B^ ABX, a basal diet supplemented with chlortetracycline; CON, basal diet; GSH1, a basal diet supplemented with 50 mg/kg GSH; GSH2, a basal diet supplemented with 65 mg/kg GSH; GSH3, a basal diet supplemented with 100 mg/kg GSH in the basal diet. HDLC, high density lipoprotein-cholesterol; LDLC, low density lipoprotein-cholesterol; ALT, alanine transaminase; AST, aspartate aminotransferase; AKP, alkaline phosphatase; ANOVA, one-way analysis of variance.

**Table 3:** Differentially redox-related proteins in the jejunal mucosa of weaned piglets.

Accession	Protein Description	Gene Name	FC	*p*-Value
GSH2 vs. CON				
A0A287BL83	Carbonyl reductase (NADPH)	NADPH	0.56	0.010
A0A5G2QH97	Voltage-dependent anion-selective channel protein 3	VDAC3	0.66	0.037
P51781	Glutathione S-transferase alpha M14	GSTAM14	0.57	0.001
A0A0K1TQQ0	Microsomal glutathione S-transferase 2	MGST2	0.41	0.019
A0A287B452	Voltage-dependent anion-selective channel protein 2	VDAC2	0.29	0.003
A0A287BGN0	Cytochrome c oxidase subunit	COX6A1	0.38	0.001
P04175	NADPH-cytochrome P450 reductase	POR	0.56	0.015
F1SDB7	Flavin-containing monooxygenase	FMO5	0.66	0.009
Q29092	Endoplasmin heat shock protein 90 kDa beta member 1	Hsp90B1	1.69	0.004
A0A5G2R0T1	Heat shock protein family A (Hsp70) member 4	HspA4	2.67	0.047
P36968	Phospholipid hydroperoxide glutathione peroxidase	GPX4	1.51	0.001
A7LKB1	NAD-dependent protein deacetylase sirtuin-1 isoform a	SIRT1	1.56	0.000
A4L7N3	Forkhead box protein O1	FOXO1	1.57	0.002
A0A287B2S5	Non-specific serine/threonine protein kinase	Akt1	1.52	0.028
GSH2 vs. ABX				
A0A287B452	Voltage-dependent anion-selective channel protein 2	VDAC2	0.46	0.015
A0A287BGN0	Cytochrome c oxidase subunit	COX6A1	0.44	0.004
A0A287BL83	Carbonyl reductase (NADPH)	NADPH	0.53	0.019
A0A5K1UL95	Aldo-keto reductase family 1, member C1	AKR1C3	0.43	0.045
P51781	Glutathione S-transferase alpha M14	GSTAM14	0.52	0.043
P81693	Low molecular weight phosphortyrosine protein phosphatase	ACP1	2.97	0.017
A0A287BP39	Cytochrome P450 2C42	CYP2C42	0.57	0.039
F1S6B7	Flavin-containing monooxygenase	FMO4	0.63	0.046
I6L6E1	Aldehyde dehydrogenase	ALDH3B1	0.66	0.046
P04175	NADPH-cytochrome P450 reductase	POR	0.60	0.039
Q29092	Endoplasmin	Hsp90B1	1.73	0.005
A0A5G2R0T1	Heat shock protein family A (Hsp70) member 4	HspA4	2.80	0.041
P36968	Phospholipid hydroperoxide glutathione peroxidase	GPX4	1.51	0.002
A7LKB1	NAD-dependent protein deacetylase sirtuin-1 isoform a	SIRT1	1.57	0.002
A4L7N3	Forkhead box protein O1	FOXO1	1.56	0.001
A0A287B2S5	Non-specific serine/threonine protein kinase	Akt1	1.52	<0.0001
ABX vs. CON				
A0A287ANH8	H (+)-transporting two-sector ATPase	ATPase	0.65	0.037
A0A287BMK6	Cytochrome P450 family 4 subfamily F member 3	CYP4F3	4.16	0.021
F1SC62	Cytochrome P450 2C42	CYP2C42	2.12	0.017
A0A287B2S5	Non-specific serine/threonine protein kinase	Akt1	0.08	<0.0001

Only proteins with VIP > 1, FC > 1.5 or FC < 0.67, and *p* < 0.05 were defined as differentiated proteins. FC, fold change. ABX, a basal diet supplemented with chlortetracycline; CON, basal diet; GSH1, a basal diet supplemented with 50 mg/kg GSH; GSH2, a basal diet supplemented with 65 mg/kg GSH; GSH3, a basal diet supplemented with 100 mg/kg GSH in the basal diet.

## Data Availability

All data are included in the article.

## Appendix A

**Table A1:** Composition and nutrient levels of diets (as-fed basis, %).

Ingredients	6–11 kg	11–25 kg
Corn	35.31	47.55
Extruded corn	15.00	13.00
Fermented soybean meal	9.00	8.50
Soybean meal	——	9.00
Extruded soybean	10.00	6.00
Fish meal	4.00	4.00
Whey powder	11.00	6.00
Soybean hull	5.00	——
Soybean oil	1.20	——
Plasma protein powder	3.00	——
White granulated sugar	2.00	2.00
50% Choline chloride	0.20	0.18
Sodium chloride	0.45	0.45
Calcium hydrophosphate	0.62	0.60
Limestone	0.65	0.74
Zinc oxide	0.30	——
Copper sulfate	——	0.015
L-Lysine	0.60	0.54
DL-Methionine	0.22	0.2
L-Threonine	0.21	0.21
L-Trptophan	0.04	0.03
Premix compound ^A^	1.50	1.00
Total	100.00	100.00
Nutrient levels ^B^		
DE (MJ/kg)	14.86	14.72
Crude protein	19.20	19.10
Total calcium	0.68	0.70
Total phosphorus	0.56	0.53
STTD phosphorus	0.39	0.34
SID Lysine	1.57	1.41
SID Methionine + Cysteine	0.89	0.81
SID Threonine	0.97	0.88
SID Trptophan	0.26	0.23

The values are expressed as percentage (%) except DE. DE: digestible energy; STTD: standard total intestinal digestibility; SID: standardized ileal digestibility. ^A^ Premix provided these amounts of vitamins and minerals per kilogram on an as-fed basis for piglets: vitamin A, 2400 IU; vitamin D3, 2800 IU; vitamin E, 200 IU; vitamin K3, 5 mg; vitamin B1, 3 mg; vitamin B2, 10 mg; vitamin B12, 40 μg; niacin, 40 mg; pantothenic acid, 15 mg; folic acid, 1 mg; vitamin B6, 8 mg; biotin, 0.08 mg; Fe, 120 mg as ferrous sulfate; Cu, 16 mg as copper sulfate; Mn, 70 mg as manganese oxide; Zn, 120 mg as zinc oxide; I, 0.7 mg as potassium iodide; and Se, 0.48 mg as sodium selenite. ^B^ Crude protein, total calcium, and total phosphorus were measured values according to the China National Standard [63,64,65]. Whereas the others were calculated values from data provided by Feed Database in China [66].

## Appendix B

**Table A2:** Primers sequences used for qRT-PCR analysis.

Gene	Primer Sequence (5′→3′)	Accession No.
*β-Actin*	Forward: CACGCCATCCTGCGTCTGGA	XM_003124280.4
	Reverse: AGCACCGTGTTGGCGTAGAG	
*ZO-*1	Forward: AGCCCGAGGCGTGTTT	XM_013993251.1
	Reverse: GGTGGGAGGATGCTGTTG	
*Claudin*1	Forward: AAATCAGAACTTTGGAGGC	NM_021101.4
	Reverse: AAACAAGAGTGCTATGGGTC	
*Occludin*	Forward: GCACCCAGCAACGACAT	NM_001163647.2
	Reverse: CATAGACAGAATCCGAATCAC	
*Muc*1	Forward: CGCCTGCCTGAATCTGTT	NM_001018016.2
	Reverse: GCTCTTGGTAGTAGTCGGTGC	
*Muc*2	Forward: CTGCTCCGGGTCCTGTGGGA	XM_007465997.1
	Reverse: CCCGCTGGCTGGTGCGATAC	
*MLCK*	Forward: CTCCAAGGACCGGATGAA	XM_001929078.6
	Reverse: CCACTGAGCCCTGAGATCAT	
*GPX*4	Forward: TGAGGCAAGACGGAGGTAAACT	NM_214407
	Reverse: TCCGTAAACCACACTCAGCATATC	
*Hsp*70	Forward: GCCCTGAATCCGCAGAATA	NM_001123127.1
	Reverse: TCCCCACGGTAGGAAACG	
*Hsp*90	Forward: AAGCCCTGAGAGACAACTCG	U94395.1
	Reverse: TGAAGCCAGAAGACAGCAGA	
*SIRT*1	Forward: GTTAGGAGGTGAATATGCCAAG	NM_001145750.2
	Reverse: CAACTCTTTTTGTGTTCGTGGA	
*FoxO*1	Forward: CCAGTCTTCACCAGGCACCA	NM_214014.3
	Reverse: GCCTCCGTAACTCGATTTGCT	
*Akt*1	Forward: TCATGCAGCACCGTTTCTT	NM_001159776.1
	Reverse: AATACCTGGTGTCCGTCTCG	
*β-Actin*	Forward: CACGCCATCCTGCGTCTGGA	XM_003124280.4
	Reverse: AGCACCGTGTTGGCGTAGAG	
*ZO-*1	Forward: AGCCCGAGGCGTGTTT	XM_013993251.1
	Reverse: GGTGGGAGGATGCTGTTG	
*Claudin*1	Forward: AAATCAGAACTTTGGAGGC	NM_021101.4
	Reverse: AAACAAGAGTGCTATGGGTC	
*Occludin*	Forward: GCACCCAGCAACGACAT	NM_001163647.2
	Reverse: CATAGACAGAATCCGAATCAC	
*Muc*1	Forward: CGCCTGCCTGAATCTGTT	NM_001018016.2

ZO-1, zonula occludens-1; Muc, mucin; MLCK, myosin light chain kinas; GPX4, glutathione peroxidase 4; Hsp70, heat shock protein 70 kDa; Hsp90, heat shock protein 90 kDa; SIRT1, sirtuin 1; FoxO1, forkhead box protein O1; Akt1, serine/threonine kinase 1.
